# Cross-species inference of long non-coding RNAs greatly expands the ruminant transcriptome

**DOI:** 10.1186/s12711-018-0391-0

**Published:** 2018-04-24

**Authors:** Stephen J. Bush, Charity Muriuki, Mary E. B. McCulloch, Iseabail L. Farquhar, Emily L. Clark, David A. Hume

**Affiliations:** 1 The Roslin Institute, University of Edinburgh, Easter Bush Campus, Edinburgh, Midlothian EH25 9RG UK; 2 Nuffield Department of Clinical Medicine, John Radcliffe Hospital, University of Oxford, Headington, Oxford, OX3 9DU UK; 3 Centre for Synthetic and Systems Biology, CH Waddington Building, Max Borne Crescent, King’s Buildings, University of Edinburgh, Edinburgh, EH9 3BF UK; 4 Translational Research Institute, Mater Research-University of Queensland, 37 Kent Street, Woolloongabba, QLD 4102 Australia

## Abstract

**Background:**

mRNA-like long non-coding RNAs (lncRNAs) are a significant component of mammalian transcriptomes, although most are expressed only at low levels, with high tissue-specificity and/or at specific developmental stages. Thus, in many cases lncRNA detection by RNA-sequencing (RNA-seq) is compromised by stochastic sampling. To account for this and create a catalogue of ruminant lncRNAs, we compared de novo assembled lncRNAs derived from large RNA-seq datasets in transcriptional atlas projects for sheep and goats with previous lncRNAs assembled in cattle and human. We then combined the novel lncRNAs with the sheep transcriptional atlas to identify co-regulated sets of protein-coding and non-coding loci.

**Results:**

Few lncRNAs could be reproducibly assembled from a single dataset, even with deep sequencing of the same tissues from multiple animals. Furthermore, there was little sequence overlap between lncRNAs that were assembled from pooled RNA-seq data. We combined positional conservation (synteny) with cross-species mapping of candidate lncRNAs to identify a consensus set of ruminant lncRNAs and then used the RNA-seq data to demonstrate detectable and reproducible expression in each species. In sheep, 20 to 30% of lncRNAs were located close to protein-coding genes with which they are strongly co-expressed, which is consistent with the evolutionary origin of some ncRNAs in enhancer sequences. Nevertheless, most of the lncRNAs are not co-expressed with neighbouring protein-coding genes.

**Conclusions:**

Alongside substantially expanding the ruminant lncRNA repertoire, the outcomes of our analysis demonstrate that stochastic sampling can be partly overcome by combining RNA-seq datasets from related species. This has practical implications for the future discovery of lncRNAs in other species.

**Electronic supplementary material:**

The online version of this article (10.1186/s12711-018-0391-0) contains supplementary material, which is available to authorized users.

## Background

Mammalian transcriptomes include many long non-coding RNAs (lncRNAs), a collective term for transcripts of more than 200 nucleotides that resemble mRNAs (many being 3′ polyadenylated, 5′ capped and spliced) but do not encode a protein product [1]. Proposed functional roles of lncRNAs include transcriptional regulation, epigenetic regulation, intracellular trafficking and chromatin remodelling (see reviews [2–9]). Some view lncRNAs as transcriptional noise [10, 11]. Full length lncRNAs are difficult to assemble: many are expressed at low levels [12], with high tissue-specificity [13, 14], at specific developmental time points (e.g. [15–17]), and with few signs of selective constraint [18, 19]. Many are also expressed transiently, and thus may be partly degraded by the exosome complex [20].

The initial recognition of lncRNAs as widespread and *bona fide* outputs of mammalian transcription was based on the isolation and sequencing of large numbers of mouse and human full-length cDNAs [21–23], many of which were experimentally validated [24] and shown to participate in sense-antisense pairs [25]. They were captured in significant numbers because the cDNA libraries were subtracted to remove abundant transcripts. More recent studies have used RNA-sequencing (RNA-seq) to assemble larger catalogues of lncRNAs [26]. Because of the power-law relationship of individual transcript abundance in mammalian transcriptomes [27], unless sequencing is carried out at massive depth, the exons of lowly-abundant transcripts (such as lncRNAs) are subject to stochastic sampling and are detected inconsistently between technical replicates of the same sample [28]. RNA-seq is also a relatively inaccurate means of reconstructing the 5′ ends of transcripts [29]. To overcome this constraint, the FANTOM Consortium supplemented RNA-seq with Cap Analysis of Gene Expression (CAGE) data, characterising—in humans—a 5′-complete lncRNA transcriptome [30].

RNA-seq libraries from multiple tissues, cell types and developmental stages are commonly pooled to maximise the number of lncRNA gene models assembled. Genome-wide surveys have expanded the lncRNA repertoire of livestock species such as cattle (18 tissues, sequenced at approximately 40 to 100 million reads each) [31], pig (10 tissues, sequenced at approximately 6 to 40 million reads each) [32], horse (8 tissues, sequenced at approximately 20 to 200 million reads each) [33] and sheep (8 tissues, sequenced at approximately 16 million reads each) [34], complementing tissue-specific lncRNA catalogues of, for example, cattle muscle [35, 36] and skin [37], and pig adipose [38, 39], liver [40] and testis [41].

The low level of lncRNA conservation (at some loci, it appears that only the act of transcription, rather than the transcript sequence itself, is functionally relevant [42]) reduces the utility of comparative analysis of the large RNA-seq datasets available from humans [30, 43] and mouse [44]. Among 200 human and mouse lncRNAs that are each characteristic of specific immune cell types, there was less than 1% sequence conservation [45].

Here we focus on more closely related species. We have generated atlases of gene expression for the domestic sheep, *Ovis aries* [46], and the goat, *Capra hircus* (manuscript in preparation). Since these two species are closely related (sharing a common ancestor less than 10 million years ago (mya) [47]) and their respective RNA-seq datasets contain many of the same tissues, it is possible to use data from one species to infer the presence of lncRNAs in the other. Cattle and humans are more distantly related to small ruminants, nevertheless they are substantially more similar than mice. We extend our approach by using existing human and cattle lncRNA datasets to identify a consensus ruminant lncRNA transcriptome, and use the sheep transcriptional atlas to confirm that candidate lncRNAs identified by cross-species inference are reproducibly expressed. The lncRNA catalogues that we have generated in the sheep and goat are of interest in themselves [48] and contribute valuable information to the Functional Annotation of Animal Genomes (FAANG) project [49, 50].

## Methods

### Sheep RNA-sequencing data

Previously, we created an expression atlas for the domestic sheep [46], using RNA-seq data that were largely collected from adult Texel × Scottish Blackface (TxBF) sheep. Experimental protocols for tissue collection, cell isolation, RNA extraction, library preparation, RNA sequencing and quality control are as previously described [46], and independently available on the FAANG Consortium website (http://www.ftp.faang.ebi.ac.uk/ftp/protocols). All RNA-seq libraries were prepared by Edinburgh Genomics (Edinburgh Genomics, Edinburgh, UK) and sequenced using the Illumina HiSeq 2500 sequencing platform (Illumina, San Diego, USA). The majority of these libraries were sequenced to a depth of more than 25 million paired-end reads per sample using the Illumina TruSeq mRNA library preparation protocol (polyA-selected) (Illumina; Part: 15031047, Revision E). A subset of 11 transcriptionally rich ‘core’ tissues (bicep muscle, hippocampus, ileum, kidney medulla, left ventricle, liver, ovary, reticulum, spleen, testes, thymus), plus one cell type under two conditions (bone marrow derived macrophages (BMDM), unstimulated and 7 h after simulation with lipopolysaccharide (LPS)), were sequenced to a depth of more than100 million paired-end reads per sample using the Illumina TruSeq total RNA library preparation protocol (rRNA-depleted) (Illumina; Part: 15031048, Revision E). The choice of ‘core’ tissues was informed by those included in previous human and mouse transcriptional atlases [51, 52], and reflect the high proportion of protein-coding genes transcribed in each species. Other samples—in particular, bone marrow derived macrophages—were included in the sheep expression atlas as a known source of novel mRNAs [53]. For characterising lncRNAs, we assume that the transcriptional diversity of protein-coding RNAs reflects the transcriptional diversity of non-coding RNAs.

Sample metadata for all tissue and cell samples are deposited in the EBI BioSamples database under submission identifier GSB-718 (https://www.ebi.ac.uk/biosamples/groups/SAMEG317052). The raw read data, as.fastq files, are deposited in the European Nucleotide Archive (ENA) under study accession PRJEB19199 (http://www.ebi.ac.uk/ena/data/view/PRJEB19199).

### Goat RNA-sequencing data

All RNA-seq libraries for goat were prepared by Edinburgh Genomics (Edinburgh Genomics, Edinburgh, UK) (as above) and sequenced using the Illumina HiSeq 4000 sequencing platform (Illumina, San Diego, USA). These libraries were sequenced to a depth of more than 30 million paired-end reads per sample using the Illumina TruSeq mRNA library preparation protocol (polyA-selected) (Illumina; Part: 15031047, Revision E). Sample metadata for all tissue and cell samples are deposited in the EBI BioSamples database under submission identifier GSB-2131 (https://www.ebi.ac.uk/biosamples/groups/SAMEG330351). The raw read data, as.fastq files, are deposited in the ENA under study accession PRJEB23196 (http://www.ebi.ac.uk/ena/data/view/PRJEB23196).

### Identifying candidate lncRNAs in sheep and goats

Previously, we described an RNA-seq processing pipeline for sheep [46]—using the HISAT2 aligner [54] and StringTie assembler [55]—for generating a uniform, non-redundant set of de novo assembled transcripts. The same pipeline was applied to the goat RNA-seq data. This produced a single file per species, merged.gtf; that is, the output of StringTie—merge, which collates every transcript model from the 54 goat assemblies (each assembly being both individual- and tissue-specific), and 429 of the 441 assemblies within the sheep expression atlas [46] (12 sheep libraries were not used for this purpose since they were replicates of pre-existing bone marrow-derived macrophage libraries, which were prepared by using an mRNA-seq rather than a total RNA-seq protocol). Not all transcript models in either GTF will be stranded. This is because HISAT2 infers the transcription strand of a given transcript by reference to its splice sites; this is not possible for single exon transcripts, which are un-spliced.

The GTF was parsed to distinguish candidate lncRNAs from assembly artefacts, and from other RNA, by applying the filter criteria of Ilott et al. [56], excluding gene models that (a) were longer than 200 bp, (b) overlapped (by more than 1 bp on the same strand) any coordinates annotated as ‘protein-coding’ or ‘pseudogene’ (these classifications are explicitly stated in the Ensembl-hosted Oar v3.1 annotation and assumed true of all gene models in the ARS1 annotation), or (c) were associated with multiple transcript models (which are more likely to be spurious). For single-exon gene models, we used a more conservative length threshold of 500 bp—the lower threshold of 200 bp could be otherwise met by a single pair of reads. We further excluded any novel gene model that was previously considered protein-coding in each species’ expression atlas (as described in [46]); these models contain an ORF encoding a peptide homologous to a ruminant protein in the NCBI nr database [46]. These criteria establish longlists of 30,677 candidate sheep lncRNAs (14,862 of which are multi-exonic) and 7671 candidate goat lncRNAs (3289 of which are multi-exonic). The sheep genome, Oar v3.1, already contains 1858 lncRNA models, of which the StringTie assembly precisely reconstructs 1402 (75%). In spite of this pre-existing support, these models were included on the sheep longlist for independent verification. The goat genome, by contrast, was annotated with a focus on protein-coding gene models [57], by consolidating protein and cDNA alignments—from exonerate [58] and tblastn [59]—with the annotation tool EVidence Modeller (EVM) [60]. Consequently, there are no unambiguous lncRNAs in the associated GTF (http://www.ftp.ncbi.nlm.nih.gov/genomes/all/GCF/001/704/415/GCF_001704415.1_ARS1/GCF_001704415.1_ARS1_genomic.gff.gz, accessed 23rd October 2017) (unlike the Ensembl-hosted sheep annotation, the goat annotation is currently only available via NCBI).

Each longlist of candidates was assessed for coding potential using three different tools: CPAT v1.2.3 [61], which assigns coding probabilities to a given sequence based on differential hexamer usage [62] and Fickett TESTCODE score [63], PLEK v1.2, a support vector machine classifier using k-mer frequencies [64], and CPC v0.9-r2 [65], which was used in conjunction with the non-redundant sequence database, UniRef90 (the Uniref Reference Cluster, a clustered set of sequences from the UniProt KnowledgeBase that constitutes a comprehensive coverage of sequence space at a resolution of 90% identity) [66, 67] (http://www.ftp.uniprot.org/pub/databases/uniprot/uniref/uniref90/uniref90.fasta.gz, accessed 18th August 2017). CPC scores putatively coding sequences positively and non-coding sequences negatively. We retained only those sequences with a CPC score less than − 0.5 (consistent with previous studies [31, 37]) and a CPAT probability higher than 0.58 (after creating sheep-specific coding and non-coding CPAT training data, from Oar v3.1 CDS and ncRNA, this cut-off is the intersection of two receiver operating characteristic curves, obtained using the R package ROCR [68]; this cut-off is also used for the goat data, as there are insufficient non-coding training data for this species).

For each remaining gene model, we concatenated its exon sequence and identified the longest ORF within it. Should CPC, CPAT or PLEK make a false positive classification of ‘non-coding’, this translated ORF was considered the most likely peptide encoded by the gene. Gene models were further excluded if the translated ORF (a) contained a protein domain, based on a search by HMMER v3.1b2 [69] of the Pfam database of protein families, v31.0 [70], with a threshold E-value of 1 × 10^−5^, or (b) shared homology with a known peptide in the Swiss-Prot March 2016 release [71, 72], based on a search with BLAST + v2.3.0 [59]: blastp with a threshold E-value of 1 × 10^−5^. Shortlists of 12,296 (sheep) and 2657 (goat) candidate lncRNAs—each with three independent ‘non-coding’ classifications and no detectable blastp and HMMER hits—are in Tables S1 and S2 [see Additional file 1: Table S1 and Additional file 2: Table S2], respectively.

### Classification of lncRNAs

Using the set of Oar v3.1 transcription start sites (TSS), which was obtained from Ensembl BioMart [73], and the set of ARS1 gene start sites (http://www.ftp.ncbi.nlm.nih.gov/genomes/all/GCF/001/704/415/GCF_001704415.1_ARS1/GCF_001704415.1_ARS1_genomic.gff.gz, accessed 23rd October 2017), we classified novel candidate lncRNAs for each species as done in [74], as either (a) sense or antisense (if the coordinates of the lncRNAs overlap, or are encapsulated by, a known gene on the same, or opposite, strand), (b) up- or downstream, and on the same or opposite strand (if < 5 kb from the nearest TSS), or (c) intergenic (if ≥ 5, 10, 20, 50, 100, 500 kb or 1 Mb from the nearest TSS, irrespective of strand). The HISAT2/StringTie pipeline, used to generate these transcript models, could not infer the transcription strand in all cases, particularly for single-exon transcripts. Accordingly, some lncRNAs will overlap the coordinates of a known gene, but its strandedness with respect to that gene—whether it is sense or antisense—will be unknown.

### Conservation of lncRNAs in terms of sequence

To assess the sequence-level conservation of sheep and goat lncRNA transcripts, we obtained human lncRNA sequences from two databases, NONCODE v5 [75] (http://www.noncode.org/datadownload/NONCODEv5_human.fa.gz, accessed 27th September 2017) and lncRNAdb v2.0 [76] (http://www.lncrnadb.com/media/cms_page_media/10651/Sequences_lncrnadb_27Jan2015.csv, accessed 27th September 2017) (which contain 172,216 and 152 lncRNAs, respectively). A previous study of lncRNAs in cattle [31] also generated a conservative set of 9778 lncRNAs, all of which were detectably expressed in at least one of the 18 tissues (read count > 25 in each of three replicates per tissue). These sets of sequences constitute three independent BLAST databases. For each sheep and goat lncRNA, blastn searches [59] were made against each database using an arbitrarily high E-value of 10, as substantial sequence-level conservation was not expected.

### Conservation of lncRNAs in terms of synteny

For each of the human (GRCh38.p10), sheep (Oar v3.1), cattle (UMD3.1) and goat (ARS1) reference genomes, we established those regions in each pairwise comparison where gene order is conserved, obtaining reference annotations from Ensembl BioMart v90 [73] (sheep, cattle and human) and NCBI (goat; http://www.ftp.ncbi.nlm.nih.gov/genomes/all/GCF/001/704/415/GCF_001704415.1_ARS1/GCF_001704415.1_ARS1_genomic.gff.gz, accessed 27th September 2017). By advancing a sliding window across each chromosome gene-by-gene from the 5′ end, we identified the first upstream and first downstream gene of each focal gene, irrespective of strand. For the purpose of this analysis, the first and last genes on each chromosome are excluded, since they have no upstream or downstream neighbour, respectively. Then, for each pairwise species comparison, we determined which sets of blocks were present in both—that is, where the HGNC symbols for upstream gene/focal gene/downstream gene were identical. These syntenic blocks, of three consecutive genes each, are regions in the genome where gene order is conserved both up- and downstream of a focal gene: between sheep and cattle, there are 2927 regions (comprising 5601 unique genes); sheep and goat, 2038 regions (3883 unique genes); cattle and goat, 2982 regions (5258 unique genes); sheep and human, 380 regions (930 unique genes); goat and human, 527 regions (1262 unique genes); cattle and human, 443 regions (1063 unique genes). If in each syntenic block a lncRNA was found between the upstream and focal gene, or the focal and downstream gene, in only one of the two species, a global alignment was made between the transcript and the intergenic region of the corresponding species. Alignments were made using the Needleman-Wunsch algorithm, as implemented by the ‘needle’ module of EMBOSS v6.6.0 [77], with default parameters. By effectively treating lncRNA transcripts as if they were CAGE tags (that is, short reads of 20 to 50 nucleotides [78]), we considered successful alignments as those containing one or more consecutive runs of 20 identical residues, without gaps. The probability that a transcript randomly matches 20 consecutive residues, within a pre-defined region, is extremely low.

For successful alignments, the target sequence (that is, an extract from the intergenic region) was considered to be a novel lncRNA. For this analysis, the sheep and goat lncRNAs used are those from their respective shortlists [see Additional file 1: Table S1 and Additional file 2: Table S2]. Locations of lncRNAs in other species are obtained from previous studies that applied similarly conservative classification criteria. For cattle, 9778 lncRNAs were obtained [31], each of which were longer than 200 bp, considered non-coding by the classification tools CPC [65] and CNCI [79], lacked sequence similarity to the NCBI nr [46] and Pfam databases [70], and had a normalised read count higher than 25 in at least two of three replicates per tissue for 18 tissues. For human, 17,134 lncRNAs were obtained [80], each of which were assembled from transfrags longer than 250 bp, considered non-coding by the classification tool CPAT [61], lacked sequence similarity to the Pfam database [70], and had active transcription confirmed by intersecting intervals surrounding the transcriptional start site with chromatin immunoprecipitation and sequencing (ChIP-seq) data from 13 cell lines.

### Quantification of expression level

For the 11 ‘core’ tissues of the sheep expression atlas, plus unstimulated and LPS-stimulated BMDMs (for details Table S2 in [46] and available under ENA accession PRJEB19199), expression was quantified using Kallisto v0.43.0 [81] with a k-mer index (k = 31) derived after supplementing the Oar v3.1 reference transcriptome with the shortlist of 11,646 novel sheep lncRNA models (Additional file 1: Table S1) and those lncRNAs assembled in either human (n = 18), goat (n = 164), or cattle (n = 1219), and which map to a conserved region of the sheep genome [see Additional file 3: Table S3]. Oar v3.1 transcripts were obtained from Ensembl v90 [73] in the form of separate files for 22,823 CDS (http://www.ftp.ensembl.org/pub/release-90/fasta/ovis_aries/cds/Ovis_aries.Oar_v3.1.cds.all.fa.gz, accessed 27th September 2017) and 6005 ncRNAs (http://www.ftp.ensembl.org/pub/release-90/fasta/ovis_aries/ncrna/Ovis_aries.Oar_v3.1.ncrna.fa.gz, accessed 27th September 2017).

An equivalent set of expression estimates was made for goat, across the 21 tissues and cell types of the goat expression atlas (i.e., 54 RNA-seq libraries available under ENA accession PRJEB23196). 47,193 transcripts, from assembly ARS1, were obtained from NCBI (http://www.ftp.ncbi.nlm.nih.gov/genomes/all/GCF/001/704/415/GCF_001704415.1_ARS1/GCF_001704415.1_ARS1_rna.fna.gz, accessed 27th September 2017), and supplemented both with the shortlist of 2657 novel goat lncRNA models [see Additional file 2: Table S2], and those lncRNAs assembled in human (n = 15), sheep (n = 507), or cattle (n = 1213) [see Additional file 3: Table S3]. After quantification in each species, transcript-level abundances were summarised to the gene-level.

### Categorisation of expression profiles

Expression levels were categorised as done in the Human Protein Atlas [82], and as previously employed in the Sheep Gene Expression Atlas [46]. Each gene is considered to have either no detectable expression (average TPM < 1, a threshold chosen to minimise the influence of stochastic sampling), low expression (10 > average TPM ≥ 1), medium expression (50 > average TPM > 10), or high expression (average TPM ≥ 50). Three sample specificity indices were calculated for each gene, as in [46]. These include *tau*, a scalar measure of expression breadth bound between 0 (for housekeeping genes) and 1 (for genes expressed in one sample only) [83], and the mean TPM (across all samples) divided by the median TPM (across all tissues). Genes with greater sample specificity will have a more strongly skewed distribution (i.e. a higher mean and a lower median), and so the larger the ratio, the more sample-specific the expression. To avoid undefined values, if median TPM is equal to 0, it is set to 0.01.

We also calculated a preferential expression measure (PEM), as in [84]. PEM is calculated per tissue per gene (unlike *tau*) and quantifies the expression of a given gene in a given tissue in relation to its average expression across all tissues (more tissue-specific genes will have higher PEM values for that tissue). For each gene *i* in tissue *t*_*i*_, PEM(*t*_*i*_) = S − A, where S = expression of gene *i* in tissue *t*_*i*_, and A = arithmetic mean expression of gene *i* across all tissues. As there are biological replicates of each tissue, we considered *S* to be the mean TPM per gene and *A* to be the mean of all values of *S*. Before PEM was calculated, all values less than 1 were considered to be 1, and a log_2_-transformation was then applied.

Each gene is also assigned one or more categories, to allow an at-a-glance overview of its expression profile: (a) ‘tissue enriched’ (expression in one tissue at least five-fold higher than all other tissues [‘tissue specific’ if all other tissues have 0 TPM]), (b) ‘tissue enhanced’ (five-fold higher average TPM in one or more tissues compared to the mean TPM of all tissues with detectable expression [this category is mutually exclusive with ‘tissue enriched’), (c) ‘group enriched’ (five-fold higher average TPM in a group of two or more tissues compared to all other tissues (‘groups’ are analogous to organ systems, and are as described in the sheep expression atlas [46]), (d) mixed expression (detected in one or more tissues and neither of the previous categories), (e) ‘expressed in all’ (more than 1 TPM in all tissues), and (f) ‘not detected’ (less than 1 TPM in all tissues).

#### Network analysis

Network analysis of the sheep expression level data was performed using Graphia Professional (Kajeka Ltd, Edinburgh, UK), a commercial version of BioLayout *Express*^3D^ [85, 86]. A correlation matrix was built for each gene-to-gene comparison, which was then filtered by removing all correlations below a given threshold (Pearson’s *r* < 0.95). A network graph was constructed by connecting nodes (genes) with edges (correlations above the threshold). The local structure of the graph—that is, clusters of co-expressed genes—was interpreted by applying the Markov clustering (MCL) algorithm [87] at an inflation value (which determines cluster granularity) of 2.2.

### Enrichment of lncRNAs in the vicinity of protein-coding genes

To test whether lncRNAs that are co-expressed with protein-coding genes are more likely to be closer to them (from which we can infer that they are more likely to have been derived from an enhancer sequence affecting that protein-coding gene), we used a randomisation test as in [88]. First, we obtained clusters of co-expressed genes from a network graph of the sheep expression level dataset (see above). We then calculated *q*, the number of times the distance between each lncRNA and the nearest protein-coding gene within the same cluster was higher than the distance between each lncRNA and the nearest gene within *s* = 1000 randomly selected, equally sized, subsets of protein-coding genes, drawn from the same chromosome as each lncRNA. Letting *r* = *s* − *q*, then the p-value of this test is *r* + 1/*s* + 1.

## Results and discussion

### Identifying lncRNAs in the sheep and goat transcriptomes

Previously, we created an expression atlas for the domestic sheep [46], using both polyadenylated and rRNA-depleted RNA-seq data that were collected primarily from three male and three female adult Texel × Scottish Blackface (T × BF) sheep at 2 years of age: 441 RNA-seq libraries in total, comprising five cell types and multiple tissues spanning all major organ systems and several developmental stages, from embryonic to adult. To complement this dataset, we also created a smaller-scale expression atlas—of 54 mRNA-seq libraries—from 6-day old crossbred goats, which will be the subject of a dedicated analysis. For both species, each RNA-seq library was aligned against its reference genome (Oar v3.1 and ARS1, for sheep and goat, respectively) using HISAT2 [54], with transcripts assembled using StringTie [55]. This pipeline produced a non-redundant set of de novo gene and transcript models, as previously described [46], and expanded the set of transcripts in each reference genome to include ab initio lncRNA predictions and novel protein-coding genes. As the primary purpose of the sheep expression atlas was to improve the functional characterisation of the protein-coding transcriptome, the novel sheep protein-coding transcript models generated by this pipeline are discussed in [46] (novel protein-coding transcripts for goats will be discussed in a dedicated analysis of the protein-coding goat transcriptome).

Using similar filter criteria to a previous study [56], the de novo gene models were parsed to create longlists of 30,677 (sheep) and 7671 (goat) candidate lncRNAs, each of which was longer than 200 bp and was not associated, on the same strand, with a known protein-coding locus. The fourfold difference in the length of each longlist can be attributed to the relative size of each dataset. The sheep atlas contains 8 times as many RNA-seq libraries, spans multiple developmental stages (from embryonic to adult), and has a subset of its samples that was specifically prepared to ensure the comprehensive capture of ncRNAs—unlike any sample in the goat dataset, this subset is sequenced at a fourfold higher depth (> 100 million reads, rather than > 25 million reads) using a total RNA-seq, rather than mRNA-seq, protocol.

Each model on both longlists was assessed for coding potential using the classification tools CPC [65], CPAT [61] and PLEK [64], alongside homology searches of its longest ORF—with blastp [59] and HMMER [69]—to known protein and domain sequences (within the Swiss-Prot [71, 72] and Pfam-A [70] databases, respectively). Those gene models classified as non-coding by CPC, CPAT and PLEK, and having no detectable blastp and HMMER hits, are considered novel lncRNAs.

This pipeline creates shortlists of 12,296 (sheep) and 2657 (goat) lncRNAs [see Additional file 1: Table S1 and Additional file 2: Table S2], respectively), representing approximately 40% (sheep) and 35% (goat) of the gene models on each longlist. The mean gene length is similar in both shortlists—6.7 kb (sheep) and 8.8 kb (goat)—as its summed exon length, averaging 1.2 kb in each species.

Consistent with previous analyses in several other species [31, 89], 6956 (57%) of the sheep lncRNAs, and 1284 (48%) of the goat lncRNAs, were single-exonic. For sheep, the shortlist contains 11,646 previously unknown lncRNA models and provides additional evidence for 650 existing Oar v3.1 lncRNA models (Additional file 1: Table S1). A small proportion of longlisted gene models were considered non-coding by at least one of CPC, CPAT or PLEK, nevertheless they showed some degree of sequence homology to either a known protein or protein domain: for sheep, 226 (including 13 existing Oar v3.1 models) [see Additional file 1: Table S4], and for goats, 153 [see Additional file 2: Table S5]. The number of novel lncRNAs identified is also given per chromosome, for sheep [see Additional file 1: Table S6] and for goat [see Additional file 2: Table S7] and per type, for sheep [see Additional file 1: Table S8] and for goat [see Additional file 2: Table S9], the majority of which—in both species—are found in intergenic regions, 10 to 100 kb from the nearest gene. Overall, the addition of these lncRNA models increases the total number of genes in the reference annotation by approximately 30% (sheep) and 12% (goat).

### The sets of ab initio sheep and goat lncRNAs only minimally overlap at the sequence level

**Fig. 1:**
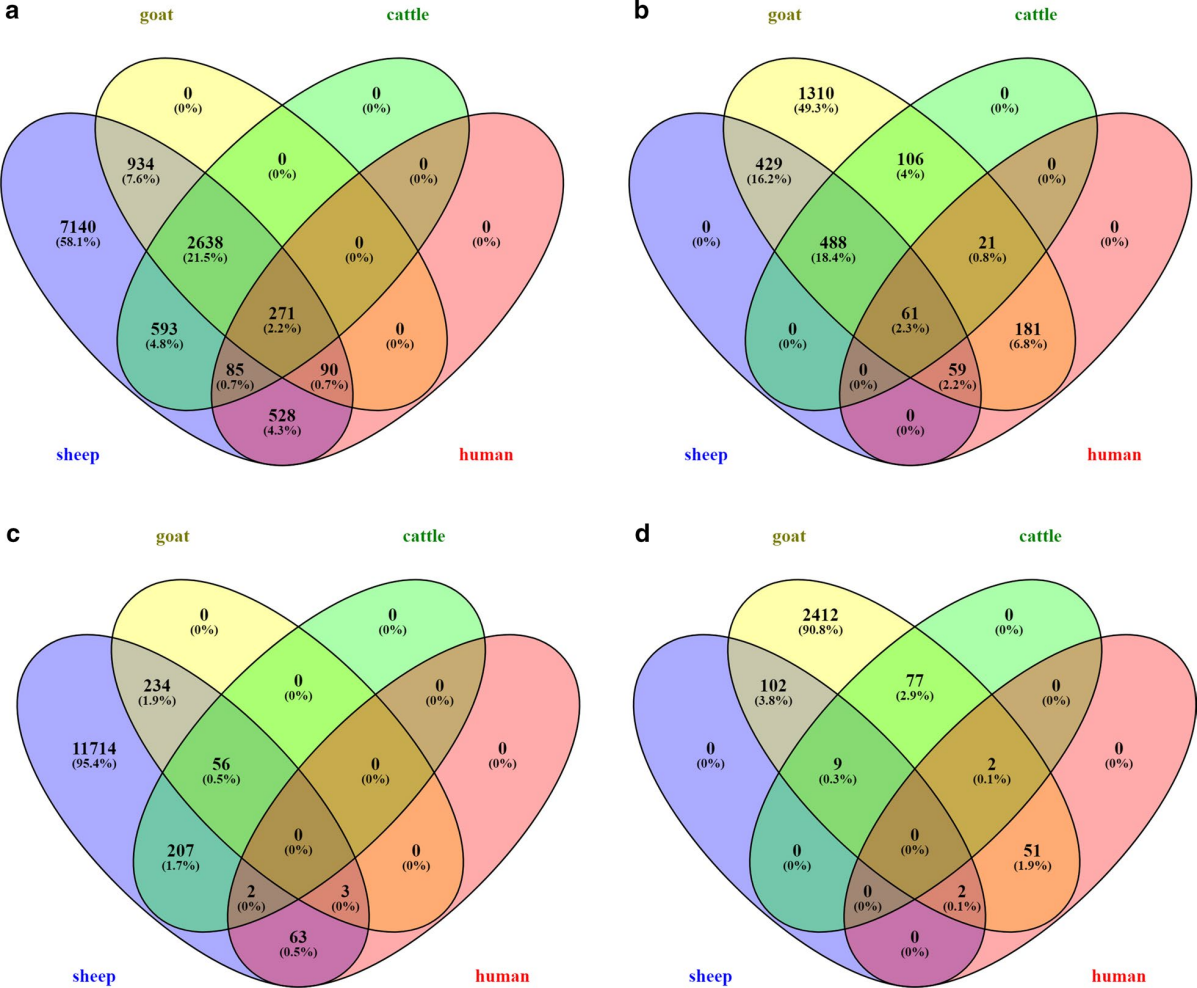
Minimal overlap of lncRNAs at the sequence level. Venn diagrams show the number of sheep (**a** and **c**) or goat (**b** and **d**) lncRNAs that can be aligned—either with an alignment of any length or quality (A and B), or with ≥ 50% identity over ≥ 50% of the length of the target sequence (**c** and **d**)—to either shortlist of goat (**a** and **c**) or sheep (**b** and **d**) lncRNAs, and to sets of cattle and human lncRNAs from previous studies. The majority (58% of sheep lncRNAs, and 49% of goat lncRNAs) have no associated alignment. Alignments are detailed in Additional file 1: Table S10 (sheep) and Additional file 1: Table S11 (goat)

Even with full length cDNA sequences, comparative analysis revealed that the majority are not conserved between species: estimates of the proportion of human lncRNAs with mouse counterparts range from 14 [90]–27% [23] (see also review [19]). When comparing the sets of sheep and goat lncRNAs, few predicted transcripts—in either species—show sequence-level similarity either to each other or to other closely or distantly related species (cattle and humans, respectively, which shared a common ancestor with sheep and goats approx. 25 and 95 mya [47]). Of the 12,296 shortlisted sheep lncRNAs, less than half (n = 5139, i.e. 42%) had any detectable pairwise alignment—of any quality and of any length—to either the shortlisted goat lncRNAs, a set of 9778 cattle lncRNAs from a previous study [31] or two sets of human lncRNAs (Fig. 1 and Table S10 [see Additional file 1: Table S10]).

High confidence is possible only for a small proportion of these alignments, i.e. the alignment has a % identity higher than 50% within an alignment longer than 50% of the length of the target sequence. Of the 5139 sheep lncRNAs that could be aligned to any species, only 293 (5.7%) could be aligned with high confidence to goat and 265 (5.2%) to cattle transcripts. Similarly, of the sheep lncRNAs that could be aligned to either of two human lncRNA databases—NONCODE [75] and lncRNAdb [76]—68 (1.6% of the total alignable lncRNA) aligned with high confidence to the NONCODE database, and none to the lncRNAdb. Similar findings are observed with the 2657 shortlisted goat lncRNAs: 1343 (50.5%) had a detectable pairwise alignment, of any quality, to either set of sheep, cattle or human lncRNAs. However, of these 1343 lncRNAs, only 113 (8.4%) aligned with high confidence to sheep, 88 (6.6%) to cattle, 55 (4.1%) to the human NONCODE database, and 1 (0.1%) to the human lncRNAdb database (Fig. 1 and Table S11 [see Additional file 2: Table S11]). These observations allow for two possibilities. First, lncRNAs may, in general, be poorly conserved at the sequence level, which is consistent with previous findings [18, 19] and the observation that only 6% of the sheep/goat alignments have more than 50% reciprocal identity. However, an alternative is that in spite of the apparent depth of coverage, we have only assembled a subset of the total lncRNA transcriptome in each species.

### lncRNAs not captured by the RNA-seq libraries of one species can be found using data from a related species

A reasonable a priori prediction is that lncRNAs conserved in a closely related species—which are more likely to be functionally relevant—are also more similarly expressed. Whereas human and mouse lncRNAs identified as full length cDNAs were generally less conserved between species than the 5′ and 3′UTR of protein-coding transcripts, their promoters were more highly conserved than those of protein-coding transcripts, some extending as far as chicken [44, 91]. These findings suggested that the large majority of lncRNAs that were analyzed displayed positional conservation across species. Accordingly, rather than comparing the similarity of two sets of lncRNA transcripts, we mapped the lncRNAs assembled in one species (e.g. sheep) to the genome of another (e.g. goat), deriving confidence in the mapping location from synteny.

For each of the pairwise sheep/cattle, sheep/goat, cattle/goat, sheep/human, goat/human, and cattle/human comparisons, we identified sets of syntenic blocks: regions in the genome where gene order is conserved both up- and downstream of a focal gene (see Table 1 and the “Methods” section).

**Table 1:** Comparatively few lncRNAs appear positionally conserved, suggesting minimal overlap between each species’ set of transcripts

Species 1	Species 2	Number of syntenic blocks (i.e. three conserved consecutive genes)	Number of unique protein-coding genes in the set of syntenic blocks	Total number of positionally conserved lncRNAs in the set of syntenic blocks (in either the up- or downstream position)	% of syntenic blocks with at least one positionally conserved lncRNA
Sheep	Cattle	2927	5601	280	9.57
Sheep	Goat	2038	3883	82	4.02
Sheep	Human	380	930	8	2.11
Goat	Cattle	2982	5258	169	5.67
Goat	Human	527	1262	2	0.38
Cattle	Human	443	1063	5	1.13

The results suggest that the lncRNAs that are expected to be found at a given genomic location are captured in only one species, not both, consistent with the stochastic sampling of lncRNAs by RNA-seq libraries

In the sheep/cattle comparison, approximately 5% of the syntenic blocks contain at least one lncRNA with a relative position conserved in both species, either upstream (n = 139 lncRNAs) or downstream (n = 141) of the central gene in each block [see Additional file 3: Table S12]. In the sheep/goat and cattle/goat comparisons, respectively, approximately 2 and 3% of the syntenic blocks contain a lncRNA (for sheep/goat, n = 42 upstream, 40 downstream; for cattle/goat, 86 upstream, 83 downstream) [see Additional file 3: Tables S13 and S14]. With increased species divergence, far fewer lncRNAs have relative positions conserved in either the upstream or downstream positions of the sheep/human, goat/human and cattle/human syntenic blocks (typically, < 1% of the syntenic blocks contain a lncRNA) [see Additional file 3: Tables S15, S16 and S17]. These comparatively small proportions highlight the minimal overlap between each set of assembled transcripts, which is consistent with stochastic assembly—lncRNAs expected to be present in a particular location are captured in only one species, not both. As such, very few lncRNAs in either of the sheep, goat and cattle subsets have evidence of both shared sequence homology and conserved synteny. When comparing sheep and cattle, 16 unique lncRNAs have high-confidence pairwise alignments within a region of conserved synteny, and six when comparing sheep and goat [see Additional file 3: Table S18].

In most of the syntenic blocks examined, if a lncRNA was detected in one location in one species (either up- or downstream of a focal gene), no corresponding assembled lncRNA was annotated in the species used for comparison, although for both species a similar range of tissues was sequenced. For example, of the 2927 syntenic blocks in the sheep/cattle comparison, 347 (12%) of the sheep blocks, and 506 (17%) of the cattle blocks, contain a lncRNA in the ‘upstream’ position (that is, between genes 1 and 2), with little overlap between the two species: in only 139 blocks (5%) is a lncRNA present in this position in both species [see Additional file 3: Table S12]. Similar results are found if the ‘downstream’ position is considered, as well as the sheep/goat, goat/cattle, sheep/human, goat/human and cattle/human comparisons: approximately 2 to 5 times as many lncRNAs are found in either of the two species than are found in both [see Additional file 3: Tables S13, S14, S15, S16 and S17].

Each set of syntenic blocks, by definition, represents a set of conserved intergenic regions. Given that the majority of the lncRNAs are intergenic [see Additional file 1: Table S8 and Additional file 2: Table S9], these regions are reasonable locations for mapping candidate transcripts (strictly speaking, concatenated exon sequences) directly to the genome. For the syntenic blocks in each species comparison, we made global alignments of the lncRNAs in species *x* to the intergenic region of species *y*, and vice versa (see Methods section). Retaining only those alignments in which the lncRNA can match the intergenic region with 20 or more consecutive residues (the majority of these alignments in any case have more than 75% identity across their entire length), we predicted 1077 additional lncRNAs in cattle, 1401 in sheep, and 1735 in goat, although only 44 in humans (Table 2).

**Table 2:** Direct mapping of lncRNA transcripts to the genome of another species

Species 1 (in which lncRNAs are captured by RNA-seq libraries)	Species 2 (in which lncRNA can be inferred)	Number of lncRNA models detected within a region of conserved synteny between species 1 and 2, but not captured by the RNA-seq libraries of species 2	Number of lncRNA models from species 1 mapped to the genome of species 2	% of lncRNA models detected by direct genome mapping	Number of intergenic regions in the syntenic blocks conserved between these two species	% of intergenic regions in which a lncRNA from species 1 is inferred in species 2
Cattle	Goat	2593	1213	46.78	5964	20.34
	Human	163	20	12.27	886	2.26
	Sheep	2939	1219	41.48	5854	20.82
Goat	Cattle	2593	286	11.03	5964	4.8
	Human	76	9	11.84	1054	0.85
	Sheep	991	164	16.55	4076	4.02
Human	Cattle	163	16	9.82	886	1.81
	Goat	76	15	19.74	1054	1.42
	Sheep	93	18	19.35	760	2.37
Sheep	Cattle	2939	775	26.37	5854	13.24
	Goat	991	507	51.16	4076	12.44
	Human	93	15	16.13	760	1.97

The results show that lncRNA transcripts assembled using the RNA-seq libraries of only one species can in many cases be directly mapped to the genome of another species, assuming the lncRNA is located within a region of conserved synteny

**Fig. 2:**
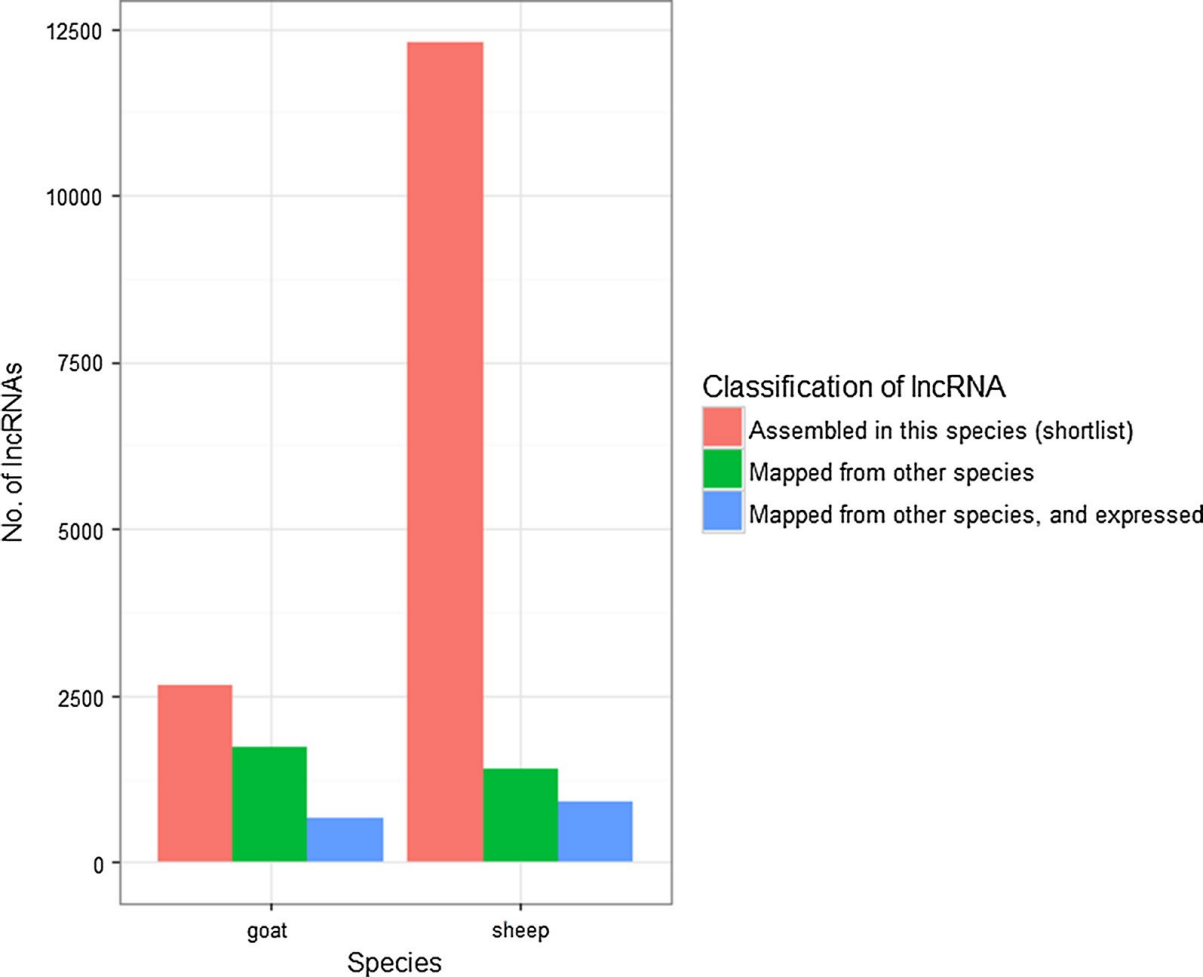
Stochastic detection and assembly of lncRNAs by RNA-seq libraries. These results—a consequence of limitations in sequencing breadth and depth—suggest that for a given species, only a subset of the total lncRNA transcriptome is likely to be captured. Nevertheless, the number of candidate lncRNAs for that species can be increased if directly mapping, to a positionally conserved region of the genome, the lncRNAs from either a related (sheep, goat, cattle) or more distant (human) species. Many of these mapped lncRNAs (which could not be completely reconstructed with the RNA-seq libraries of that species) are nevertheless detectably expressed

The fact that comparatively few ruminant lncRNAs are recognisable at the sequence level in humans (and vice versa) is consistent with the rapid turnover of the lncRNA repertoire between species [92]. In the case of the goat, the number of new lncRNAs predicted by this approach is 50% more than the number captured (and shortlisted) using goat-specific RNA-seq (Fig. 2).

This suggests that for the purposes of lncRNA detection, datasets from related species can help overcome limitations of sequencing breadth and depth. This is even apparent with comparatively large datasets—the sheep RNA-seq, for instance, spans more tissues and developmental stages than goat, but in absolute terms, it still fails to generate assemblies of many lncRNAs.

### Many of the sheep lncRNAs inferred by synteny—which could not be fully assembled from the RNA-seq reads: are nevertheless detectably expressed

To determine the expression level of the sheep lncRNAs, we used a subset of 71 high-depth (> 100 million reads) RNA-seq libraries from the sheep expression atlas [46]. This subset constitutes a set of 11 transcriptionally-rich tissues (bicep muscle, hippocampus, ileum, kidney medulla, left ventricle, liver, ovary, reticulum, spleen, testes, thymus), plus one cell type under two conditions (bone marrow derived macrophages, unstimulated and 7 h after simulation with lipopolysaccharide), each of which was sequenced in up to six individuals (where possible, three adult males and three adult females).

For each sample, expression was quantified—as transcripts per million (TPM)—using the quantification tool Kallisto [81] [see Additional file 1: Table S19]. Kallisto quantifies expression by matching k-mers from the RNA-seq reads to a pre-built index of k-mers, derived from a set of reference transcripts. For sheep, we supplemented the complete set of Oar v3.1 reference transcripts (n = 28,828 transcripts, representing 26,764 genes) both with the shortlist of 11,646 novel lncRNAs (each of which is a single-transcript gene model) [see Additional file 1: Table S1], and those lncRNAs assembled from either human, goat and cattle (respectively, 18, 164 and 1219 lncRNA; see Table 2), the presence of which was predicted in sheep by mapping the transcript to a conserved genomic region.

Of these 13,047 novel lncRNAs, 8826 were detected at a level of TPM higher than 1 in at least one of the 71 adult samples, including 14 of the human transcripts (78%), 128 of the goat transcripts (78%), and 772 of the cattle transcripts (63%) [see Additional file 1: Table S19]. At a depth of coverage of 100 million reads, we would expect to detect transcripts reproducibly at between 0.01 and 0.1 TPM if they are expressed in all the libraries that are derived from the same tissue/cell type. Indeed, of the 13,047 total novel lncRNAs, 5353 (41%) were detected with at least one paired-end read in all six replicates of the tissue in which it is most highly expressed [see Additional file 1: Table S19]. Those lncRNAs derived from goat and cattle transcripts are similarly reproducible: 83 (51%) of the goat transcripts were detected with at least one paired-end read in all 6 replicates of its most expressed tissue, as were 570 (47%) of the cattle transcripts, and 7 (39%) of the human transcripts [see Additional file 2: Table S20].

By extension, we can consider sheep, cattle and human lncRNAs to be goat lncRNAs, and create a Kallisto index containing candidate lncRNAs that are extracted from the goat genome after mapping sheep and cattle transcripts. Using such a Kallisto index (which contains the 2657 shortlisted goat lncRNAs [see Additional file 2: Table S2], 507 sheep lncRNAs, 1213 cattle lncRNAs, and 15 human lncRNAs), 1478 (34%) of a total set of 4392 candidate goat lncRNAs were reproducibly detected (> 0.01 TPM) in all four sampled goats [see Additional file 2: Table S20]. Hence, data from the sheep expression atlas can be used to provide additional functional annotation of the goat genome, in spite of the much smaller number of tissue samples compared to sheep.

In general, lncRNA expression is low: 12,325 sheep lncRNAs (94% of the total) have a mean TPM less than 10 across all 71 samples. The mean and median maximum TPM for each lncRNA across the total sheep dataset was 18.4 and 2.2 TPM, respectively [see Additional file 1: Table S19]). Other reports have described pervasive, but low-level, mammalian lncRNA transcription [12], and—given that the mean TPM exceeds the median—a high degree of lncRNA tissue-specificity [93–95]. Indeed, for those lncRNAs detected at a TPM less than 1, the average value of *tau*—a scalar measure of expression breadth bound between 0 (for housekeeping genes) and 1 (for genes expressed in one sample only) [83] (see Methods section)—is 0.66. Although most of the lncRNAs (n = 4972, 65% of the 7627 lncRNAs with an average TPM higher than 1 in at least one tissue) have idiosyncratic ‘mixed expression’ profiles (see Methods section), 1339 lncRNAs (17%) are nevertheless detected at an average TPM higher than 1 in all 13 tissues [see Additional file 1: Table S19]. Many are enriched in specific tissues, with 905 (12%) lncRNAs exhibiting either a testes-enriched (that is, fivefold higher expression in testes than other tissues) or testes-specific expression pattern (that is a TPM higher than 1 in the testes and equal to 0 in all other tissues), which is consistent with a previous study that identified numerous lncRNAs involved in ovine testicular development and spermatogenesis [96]. Many lncRNAs are expressed most strongly, even if not uniquely, in the testes. Each gene can be associated with a tissue in which it has the highest preferential expression measure (PEM; see Methods section). For the majority of genes, this tissue is the testes (n = 3770, 49% of the 7627 lncRNA with an average TPM higher than 1 in at least one tissue) [see Additional file 1: Table S19].

### Few lncRNAs are fully captured by biological replicates of the same RNA-seq library

In the largest assembly of predicted lncRNAs, from humans, the transfrags (transcript fragments) assembled from 7256 RNA-seq libraries were consolidated into 58,648 candidate lncRNAs [80]. Before assembling transfrags, machine-learning methods were used to filter, from each library, any library-specific background noise (genomic DNA contamination and incompletely processed RNA). Then, filtered libraries were merged before assembling the final gene models, which in effect is equivalent to pooling together transfrags (which may be partial or full-length transcripts) from all possible libraries. Consequently, a given set of transfrags can be assembled into a consensus transcript for a lncRNA, but that consensus transcript might not actually exist in any one cellular source. The only unequivocal means to confirm the full-length expression would be to clone the full-length cDNA. However, additional confidence could be obtained by increasing the depth of coverage in the same tissue/cell type in a technical replicate. In the sheep expression atlas, 31 diverse tissues/cell types were sampled in each of six individual adults (three females, three males, all unrelated virgin animals approximately 2 years old). By taking a subset of 31 common tissues per individual, each of the six adults was represented by ~ 0.75 billion reads.

In a typical lncRNA assembly pipeline, read alignments from all individuals are merged, to maximise the number of candidate gene models (using, for instance, StringTie—merge; see Methods). With *n* = 6 adults (and ~ 0.75 billion reads per adult), there are 2^*n*^ − 1 = 63 possible combinations of data for which GTF can be made with StringTie—merge. The reproducibility of each shortlisted lncRNA, in terms of the number of GTF it is reconstructed in, is shown in Table S21 [see Additional file 1: Table S21]. The GTF themselves are available via the University of Edinburgh DataShare portal; http://dx.doi.org/10.7488/ds/2284.

**Fig. 3:**
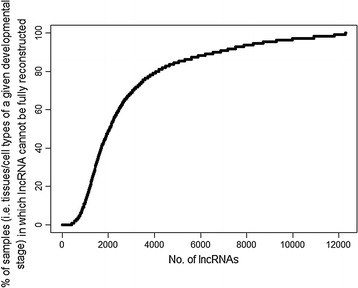
Proportion of sheep expression atlas samples for which a candidate lncRNA cannot be fully reconstructed. The sheep expression atlas comprises 429 RNA-seq libraries, representing 110 distinct samples; that is, each sample is a tissue/cell type at a given developmental stage, with up to six replicates per sample. Twenty-two candidate lncRNAs cannot be reconstructed in any given sample (i.e., the proportion of samples is 100%). These lncRNAs could be assembled only after pooling data from multiple samples. Data for this figure are in Additional file 1: Table S22

Only 812 of the 12,296 sheep lncRNAs (6.6%) could be fully reconstructed by any of the 63 GTF combinations [see Additional file 1: Table S21]. One caveat in this assessment is that these sheep libraries are exclusively from adults. Many of the 12,296 lncRNA models may instead be expressed during embryonic development. There is evidence of extensive embryonic lncRNA expression in humans [15, 97] and mouse [16, 98]. The lack of embryonic tissues could also explain why fewer lncRNAs were assembled in goat. Nevertheless, when considering all 429 RNA-seq libraries in the sheep expression atlas (i.e. including non-adult samples), there are only, on average, 29 libraries (7%) in which any individual lncRNA can be fully reconstructed (Fig. 3 and Additional file 1: Table S22).

In many cases, full-length sheep lncRNAs cannot be reconstructed using all the reads sequenced from a given individual. For instance, the known lncRNA ENSOARG00000025201 is reconstructed by 28 of the 63 possible GTF, but none of these GTF was built using reads from only one individual [see Additional file 1: Table S21]. Only 189 lncRNAs (1.5%) were fully reconstructed in all 63 possible GTF. Notably, 154 of these are known Ensembl lncRNAs [see Additional file 1: Table S21].

### lncRNAs are enriched in the vicinity of co-expressed protein-coding genes

Enhancer sequences positively modulate the transcription of nearby genes (see reviews [99, 100]), and may be the evolutionary origin of a fraction of these lncRNAs (as suggested by [101, 102]), including a novel class of enhancer-transcribed ncRNAs, enhancer RNAs (eRNAs), which—although a distinct subset—are arbitrarily classified as lncRNAs [103]. eRNAs are likely to be co-expressed with protein-coding genes in their immediate genomic vicinity.

**Fig. 4:**
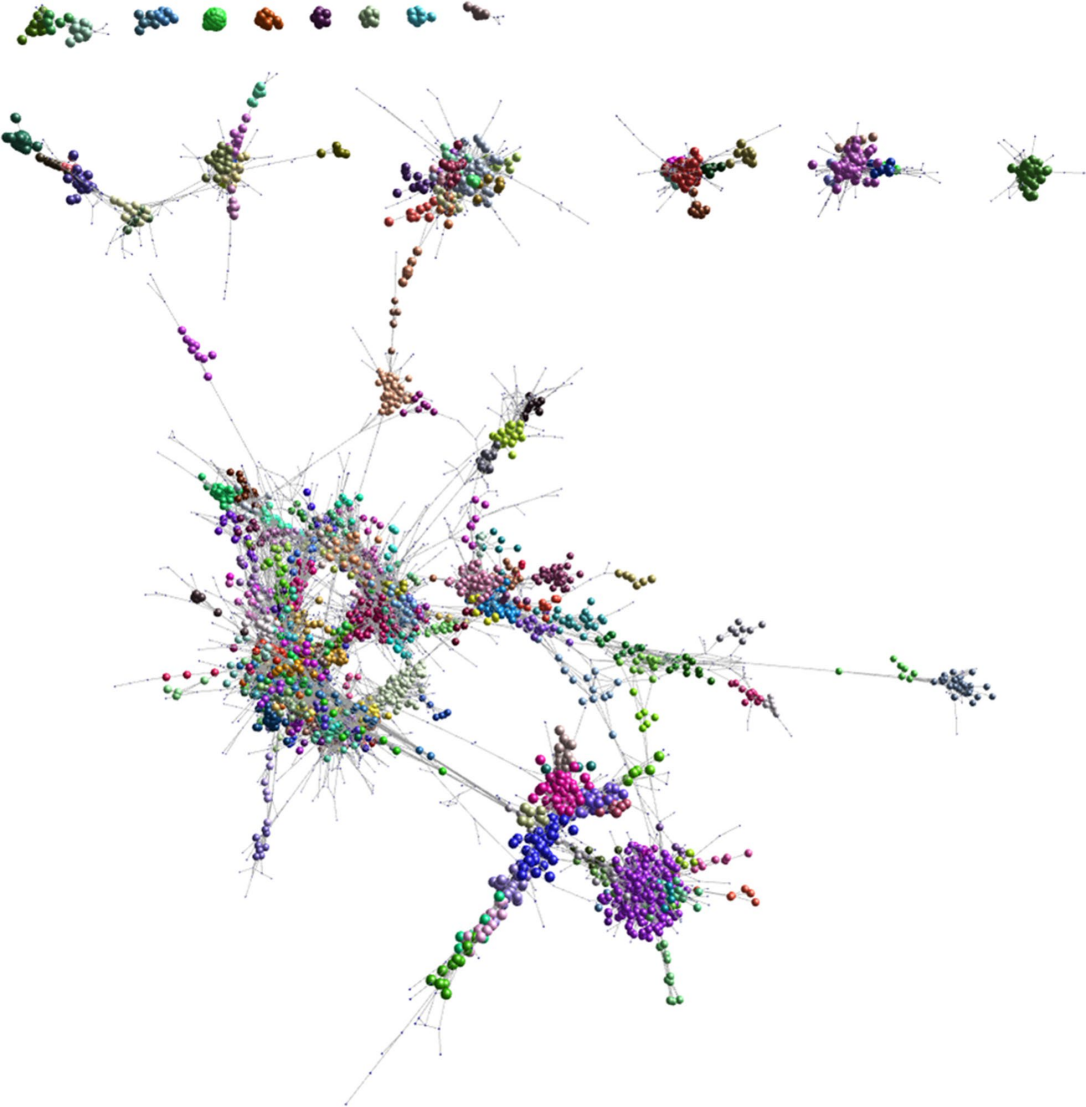
3D visualisation of a gene-to-gene correlation graph. Each node (sphere) represents a gene. Nodes are connected by edges (lines) that represent Pearson’s correlations between the two sets of expression level estimates, at a threshold greater than or equal to 0.95. The graph comprises 11,841 nodes and 2214,099 edges. Genes cluster together according to the similarity of their expression profiles (i.e. their degree of co-expression), with clusters (coloured sets of nodes) determined by using the MCL algorithm. Expression level estimates for the lncRNAs in this graph are in Additional file 1: Table S19. The genes comprising each co-expression cluster are in Additional file 1: Table S23. The lncRNAs that are co-regulated with protein-coding genes are found within the same co-expression cluster

To identify co-regulated sets of protein-coding and non-coding loci, we performed network cluster analysis of the sheep expression level dataset [see Additional file 1: Table S19] using the Markov clustering (MCL) algorithm [87], as implemented by Graphia Professional (Kajeka Ltd., Edinburgh, UK) (see Methods section) [85, 86]. To reduce noise, only those novel lncRNAs with reproducible expression (i.e. that have a TPM higher than 0.01 in every replicate of the tissue in which it is most highly expressed) are included in this analysis (n = 5353). The resulting graph contained only genes with tightly correlated expression profiles (Pearson’s *r* ≥ 0.95) (Fig. 4) and was highly structured, organised into clusters of genes with a tissue or cell-type specific expression profile [see Additional file 1: Table S23].

We expect that for a given cluster of co-expressed genes (which contains *x* lncRNAs and *y* protein-coding genes, each on chromosome *z*), the distance between an enhancer-derived lncRNA and the nearest protein-coding gene should be significantly shorter than the distance between that lncRNA and a random subset of protein-coding genes. For the purposes of this test, each random subset, of size *y*, is drawn from the complete set of protein-coding genes on the same chromosome *z* (that is, the same chromosome as the lncRNA), irrespective of strand and their degree of co-expression with the lncRNA. The significance of any difference in distance was then assessed using a randomisation test (see Methods section).

Of the 5353 lncRNAs included in the analysis, 1351 (25%) were found on the same chromosome as a highly co-expressed protein-coding gene [see Additional file 1: Table S24], with 252 of these (19%) significantly closer to the co-expressed gene than to randomly selected genes from the same chromosome (*p* < 0.05) [see Additional file 1: Table S25].

Even when the lncRNA is reproducibly expressed in each of the six animals, there is still substantial noise in the expression estimates which compromises co-expression analysis. We therefore calculated the Pearson’s *r* correlation between the expression profile of each reproducibly expressed lncRNA and its nearest protein-coding gene (which may overlap it), located both 5′ and 3′ on the sheep genome [see Additional file 1: Table S26]. The distance to the nearest gene correlates negatively with the absolute value of Pearson’s *r*, both for genes upstream (*rho* = − 0.19, *p* < 2.2 × 10^−16^) and downstream (*rho* = − 0.21, *p* < 2.2 × 10^−16^) of the lncRNA [see Additional file 1: Table S26]. This suggests that, in general, the expression profile of a lncRNA is more similar to nearer than more distant protein-coding genes. Using a variant of the above randomisation test, we also tested whether the absolute value of Pearson’s *r*, when correlating the expression profiles of the lncRNA and its nearest protein-coding gene, was significantly greater than the value of *r* obtained when correlating the lncRNA with 1000 random protein-coding genes drawn from the same chromosome. For this test, analysis was restricted to the lncRNA that were drawn from complete chromosomes rather than the smaller unplaced scaffolds. 27% of lncRNAs had a Pearson *r* higher than 0.5 with either the nearest upstream or downstream gene, and in around 20% of cases, *r* was significantly different (*p* < 0.05) from the average correlation with the random set [see Additional file 1: Table S26].

There are, in total, 2 × 6720 lncRNA/protein-coding gene pairs (i.e., the lncRNA both with its nearest up- and nearest downstream gene, excluding 491 lncRNAs on unplaced scaffolds, for which pairing was not possible) × 13 tissues/cells in which the expression of each gene is assessed. Of these 174,720 possible observations, there are 45,959 instances (26%) in which the lncRNA and the nearest protein-coding gene are both detectably expressed (TPM higher than 1), of which the largest number are found in the testes (n = 2477 lncRNAs) and hippocampus (n = 2393) [see Additional file 1: Table S26]. In conjunction with a generally closer proximity to co-expressed protein-coding genes, this further suggests there is biological relevance for particular lncRNA/mRNA pairings, particularly in these tissues.

## Conclusions

Comparative analysis of lncRNAs that are assembled using RNA-seq data from several closely related species—sheep, goat and cattle—demonstrates that the de novo assembly of lncRNAs requires very high-depth RNA-seq datasets with a large number of replicates (more than six replicates per sample, each sequencing with many more than 100 million reads). The transcription of many lncRNAs that are identified by this cross-species approach is conserved, which is a reasonable confirmation of their existence. We identified a subset of lncRNAs in close proximity to protein-coding genes with which they are strongly co-expressed, which is consistent with the evolutionary origin of some ncRNAs in enhancer sequences. Conversely, the majority of lncRNAs are not co-expressed with neighbouring protein-coding genes. Overall, alongside substantially expanding the lncRNA repertoire for several livestock species, we demonstrate that the conventional approach to lncRNA detection—that is, species-specific de novo assembly—can be reliably supplemented by data from related species.

## Additional files

**Additional file 1.** This file contains all supplementary tables relating to the characterisation of sheep lncRNAs. **Table S1.** Candidate sheep lncRNAs: a shortlist of novel gene models (plus independently confirmed known gene models) assessed for coding potential using CPC, CPAT, PLEK, blastp vs. Swiss-Prot, and HMMER vs. Pfam. **Table S4.** Sheep gene models considered as non-coding by either CPC, CPAT or PLEK but showing sequence homology to either a known protein (in Swiss-Prot) or protein domain (in Pfam-A). **Table S6.** Number of novel sheep lncRNA gene models identified per chromosome. **Table S8.** Number of novel sheep lncRNA gene models identified, by category. **Table S10.** Alignments of novel sheep lncRNA gene models to goat, cattle and human lncRNA. **Table S19.** Expression level estimates for 13,047 novel sheep lncRNAs, as transcripts per million (TPM), which were assessed using 71 adult RNA-seq libraries (11 tissues plus one cell type under two conditions, each sample is sequenced for six individuals). **Table S21.** Reproducibility of sheep lncRNA gene models when merging all combinations of data from six adults (three females and three males), for each individual a common set of RNA-seq libraries (comprising 31 tissues/cell types) was available. **Table S22.** Number of sheep expression atlas RNA-seq libraries (out of 429 in total) for which a candidate lncRNA gene model cannot be fully reconstructed. **Table S23.** Genes within each co-expression cluster, after network analysis of the sheep RNA-seq libraries. **Table S24.** Number of sheep lncRNAs co-expressed with protein-coding genes. **Table S25.** Distance between lncRNAs and protein-coding genes within the same co-expression cluster, on the same chromosome, for sheep. **Table S26.** Correlation between the expression profile of sheep lncRNAs and their nearest protein-coding genes, both 5′ and 3′.

**Additional file 2.** This file contains all supplementary tables relating to the characterisation of goat lncRNAs. **Table S2.** Candidate goat lncRNAs: a shortlist of novel gene models assessed for coding potential using CPC, CPAT, PLEK, blastp vs. Swiss-Prot, and HMMER vs. Pfam. **Table S5.** Goat gene models considered non-coding by either CPC, CPAT or PLEK but showing sequence homology to either a known protein (in Swiss-Prot) or protein domain (in Pfam-A). **Table S7.** Number of novel goat lncRNA gene models identified per chromosome. **Table S9.** Number of novel goat lncRNA gene models identified, by category. **Table S11.** Alignments of novel goat lncRNA gene models to sheep, cattle and human lncRNA. **Table S20.** Expression level estimates for 4392 novel goat lncRNAs, as transcripts per million (TPM), which were assessed using 54 RNA-seq libraries (20 tissues plus one cell type under two different conditions, for each sample four individuals were sequenced).

**Additional file 3.** This file contains all supplementary tables relating to lncRNA identification via the conservation of synteny. **Table S3**. lncRNAs inferred in one species by the genomic alignment of a transcript assembled with the RNA-seq libraries from a related spdecies. **Table S12.** Presence of intergenic lncRNAs both in sheep and cattle, in regions of conserved synteny. **Table S13.** Presence of intergenic lncRNAs both in sheep and goat, in regions of conserved synteny. **Table S14.** Presence of intergenic lncRNAs both in cattle and goat, in regions of conserved synteny. **Table S15.** Presence of intergenic lncRNAs both in sheep and humans, in regions of conserved synteny. **Table S16.** Presence of intergenic lncRNAs both in goat and humans, in regions of conserved synteny. **Table S17.** Presence of intergenic lncRNAs both in cattle and humans, in regions of conserved synteny. **Table S18.** High-confidence lncRNA pairs, those conserved across species both sequentially and positionally.
